# Further intracellular proteins and signaling pathways regulated by angiotensin-(1–7) in human endothelial cells

**DOI:** 10.1016/j.dib.2016.12.004

**Published:** 2016-12-08

**Authors:** Christian Meinert, Franziska Kohse, Ilka Böhme, Florian Gembardt, Anja Tetzner, Thomas Wieland, Barry Greenberg, Thomas Walther

**Affiliations:** a Institute of Experimental and Clinical Pharmacology and Toxicology, Medical Faculty Mannheim, Universität Heidelberg, Mannheim, Germany; b Department of Pharmacology and Therapeutics, University College Cork, Cork, Ireland; c Departments of Obstetrics and Pediatric Surgery, Division of Women and Child Health, Universität Leipzig, Leipzig, Germany; d Division of Nephrology, Department of Internal Medicine III, Faculty of Medicine, Technische Universität Dresden, Dresden, Germany; e Division of Cardiology, University of California, San Diego, USA

**Keywords:** Angiotensin-(1-7), Antibody microarray, Cell signaling, Endothelium, Renin-Angiotensin system

## Abstract

In 2016, Meinert et al. (doi: 10.1016/j.jprot.2015.09.020) published the first 25 proteins in a protein array regulated in Human Umbilical Vein Endothelial Cells (HUVEC) by the heptapeptide angiotensin (Ang)-(1–7) and the first 10 intracellular signaling cascades at different time points. This supporting data article shows further proteins and pathways stimulated by Ang-(1–7) in human endothelial cells at time points of 1 h, 3 h, 6 h, and 9 h. HUVECs were stimulated with Ang-(1–7), and regulated proteins were identified via antibody microarray. Bioinformatics software IPA was used for association of regulated proteins to metabolic pathways.

**Specifications Table**

**Table:** 

Subject area	*Cardiovascular*
More specific subject area	*Renin-angiotensin system*
Type of data	*5 tables*
How data was acquired	*Antibody microarray for regulated proteins using a GenePix 4100A Microarray Scanner (Molecular Devices, Sunnyvale, USA), and the program IPA (Ingenuity Systems, Redwood City, USA) for the identification of potential metabolic pathways.*
Data format	*analyzed*
Experimental factors	*Human Umbilical Vein Endothelial Cells were stimulated with angiotensin-(1–7)*
Experimental features	*Screening of proteins and pathways in angiotensin-(1–7) stimulated Human Umbilical Vein Endothelial Cells*
Data source location	*Cork, Ireland*
Data accessibility	*Data within this article*

**Value of the data**

• First screening of 725 proteins potentially regulated by angiotensin (Ang)-(1–7) in endothelial cells via antibody microarray.
• As often slightly regulated proteins have already dramatic biological effects, identification of further proteins altered by Ang-(1–7) might have significant scientific relevance.
• Detailed description of Ang-(1–7) effects on intracellular signaling pathways under non-pathophysiological circumstances can identify further areas of benefit using Ang-(1–7).
• The understanding of intracellular network signaling initiated by Ang-(1–7) might allow conclusions on how the heptapeptide can oppose the effects of the detrimental Ang II.

## 1 Data

The antibody microarray identified 110 regulated proteins in human umbilical vein endothelial cells (HUVEC) cells after 1-h stimulation with Ang-(1–7), 119 after 3 h, 31 after 6 h, and 86 after 9 h. The first 25 regulated proteins have been published in Meinert et al*.* [1] in Table 1, Table 2, Table 3, Table 4. Here the name and ranking of the next regulated proteins are shown (Table 1, Table 2, Table 3, Table 4). Additionally, further intracellular pathways affected by Ang-(1–7) are shown in Table 5A–D.

## 2 Experimental design, materials and methods

### 2.1 Cell culture and cell stimulation

HUVEC were grown on 100-mm dishes in EBM (Endothelial basal medium)-2 medium under standard conditions of 37 °C in a humidified incubator and 5% CO_2_ [2]. Cells were used in passage 6. When they reached 70% confluence, they were washed twice with DPBS (Dulbecco׳s phosphate-buffered saline) and serum starved for 1 h in supplements-free medium. HUVECs were stimulated with 10^−7^ M Ang-(1–7) for 1 h, 3 h, 6 h and 9 h. Control cells were treated only with DPBS (solvent).

### 2.2 Antibody microarray

After Ang-(1–7) stimulation, 1 mg/ml protein cell extract was labeled with Cy™3 or Cy™5 dye. The antibody microarray was performed as described in the Panorama Antibody Microarray-XPRESS Profiler725 Kit manual (Sigma-Aldrich, St. Louis, USA). After incubation with the labeled samples, washing and air drying images were acquired using GenePix 4100A Microarray Scanner (Molecular Devices, Sunnyvale, USA). Data was imported into Acuity 4.0 software (Molecular Devices, Sunnyvale, USA) and normalized using the nonlinear Lowess normalization method. Association of regulated proteins to metabolic pathways was done by IPA software (Ingenuity Systems, Redwood City, USA). The software calculated a *p*-value using the right tailed Fisher Exact test. The *p*-value gives the probability that the association between regulated detected proteins and the pathways is due to random association. The software considers a *p*-value <0.05 as statistically significant.

## Figures and Tables

**Table 1:** The proteins ranked 26–100 based on the detected fold change values after 1 h incubation of HUVEC with 10^−7^ M Ang-(1–7). The order of the numbers is oriented on the highest single value. Expression fold change lower than 1.5 is given in hyphen. Data that could not be detected is marked as n.d. Proteins marked in Italic show repeatedly identified differentially expressed proteins (RIDEPs). The mentioned dye indicates with which dye the unstimulated sample was labeled with.

	**Protein**	**Antibody id**	**Cy3**	**Cy5**	**Cy5**
**26.**	**CIN85**	**C8116**	**2.68**	–	–
**27.**	**Annexin VII**	**A4475**	–	**2.64**	–
**28.**	**AOP1**	**A7674**	**2.44**	**2.64**	**n.d**
**29.**	**RALAR**	**R8529**	**2.63**	**2.03**	–
**30.**	**PINCH-1**	**P9371**	–	**2.61**	–
**31.**	**Rab9**	**R5404**	**2.6**	**1.76**	–
**32.**	**BOB.1/OBF.1**	**B7810**	**2.57**	–	**n.d**
**33.**	**GFI1**	**G6670**	–	**2.51**	–
**34.**	**MAPK14 (NonActivated)**	**M8432**	**2.5**	**2.09**	–
**35.**	**Bim**	**B7929**	**2.49**	–	**n.d**
**36.**	**PSF**	**P2860**	**2.45**	**1.57**	–
**37.**	**ERK2**	**M7431**	**2.44**	**1.69**	–
**38.**	**ASPP2**	**A4480**	**1.64**	**2.44**	**n.d**
**39.**	**BACE1**	**B0806**	**2.43**	**2.29**	–
**40.**	**MTBP**	**M3566**	**2.43**	**1.79**	–
**41.**	**RICK**	**R9650**	**2.43**	**1.66**	–
**42.**	**Bmf**	**B1684**	**1.53**	**2.38**	–
**43.**	**MADD**	**M5683**	–	**2.37**	–
**44.**	**c-Raf (pSer621)**	**R1151**	**1.95**	**2.36**	**n.d**
**45.**	**p53R2**	**P4993**	**2.35**	**1.54**	–
*46.*	*SLIPR/MAGI-3*	*S4191*	*2.35*	*1.77*	–
*47.*	*SMAD4*	*S3934*	*2.30*	–	–
**48.**	**ASC-2**	**A5355**	**1.59**	**1.61**	**2.28**
**49.**	**Calretinin**	**C7479**	**2.25**	**1.96**	**n.d**
**50.**	**TBP**	**T1827**	**n.d**	**n.d**	**2.24**
**51.**	**PRNP**	**P5999**	**2.23**	–	**n.d**
*52.*	*SIAH2*	*S7945*	*2.23*	–	–
**53.**	**MTA2**	**M7569**	**2.18**	**1.56**	**1.62**
**54.**	**FAK (pTyr397)**	**F7926**	**1.73**	–	**2.17**
**55.**	**Nuf2**	**N5287**	**2.16**	–	–
**56.**	**UCHL1**	**U5258**	**2.16**	–	–
**57.**	**NBS1**	**N9287**	**2.16**	–	–
**58.**	**FKHRL1**	**F2178**	**1.74**	**2.15**	–
**59.**	**RAIDD**	**R5275**	**2.15**	–	**n.d**
**60.**	**Cyclin A**	**C4710**	**n.d**	**2.14**	–
**61.**	**BAP1**	**B9303**	–	**2.14**	**n.d**
**62.**	**p57kip2**	**P2735**	–	**2.13**	–
**63.**	**Importin α1**	**I9658**	**2.12**	–	–
**64.**	**WAVE**	**W0392**	**2.10**	–	–
**65.**	**Caldesmon**	**C6542**	**2.08**	**1.64**	–
**66.**	**AP1**	**A5968**	**2.08**	**1.60**	–
**67.**	**Zip Kinase**	**Z0134**	**2.07**	**1.66**	**n.d**
**68.**	**FLIPγ/δ**	**F9925**	**2.07**	–	–
**69.**	**ARC**	**A8344**	–	–	**2.05**
**70.**	**Fas**	**F4424**	**2.04**	**1.54**	–
**71.**	**Pan Cytokeratin**	**C2931**	**1.92**	–	**2.03**
**72.**	**S100**	**S2532**	**2.00**	**1.68**	–
**73.**	**H3 (Ac-Lys9, pSer10)**	**H0788**	–	**2.00**	–
**74.**	**H3 (Ac-Lys9)**	**H0913**	**1.99**	**1.52**	–
**75.**	**Nitrotyrosin**	**N0409**	**1.98**	**1.50**	–
**76.**	**PID/MTA2**	**P5118**	**1.98**	–	**n.d**
**77.**	**APRIL**	**A1726**	**1.91**	**1.95**	–
**78.**	**δ-Catenin/NPRAP**	**C4864**	**1.95**	–	–
**79.**	**hABH2**	**A8228**	**1.94**	**1.57**	–
**80.**	**Desmosomal Protein**	**D1286**	**1.94**	–	–
**81.**	**Coilin**	**C1862**	**1.89**	**1.94**	–
**82.**	**H3 (diMe-Lys9)**	**D5567**	–	–	**1.94**
**83.**	**TRF1**	**T1948**	**1.92**	**1.52**	–
**84.**	**cAbl**	**A5844**	**1.91**	–	–
**85.**	**Tyrosin Hydrolase**	**T2928**	**1.91**	**1.59**	–
**86.**	**β-COP**	**G6160**	**1.91**	–	**n.d**
**87.**	**E2F1**	**E9026**	**1.77**	**1.85**	–
**88.**	**SUMO1**	**S5446**	–	**1.85**	–
**89.**	**Parkin**	**P6248**	**1.85**	–	–
**90.**	**SynCAM**	**S4945**	**1.60**	**1.84**	–
**91.**	**Protein Kinase Cβ2**	**P2584**	**n.d**	**n.d**	**1.84**
**92.**	**MTA1**	**M1320**	**1.84**	–	–
**93.**	**BUB1**	**B0561**	**1.82**	**1.64**	–
**94.**	**ASPP1**	**A4355**	**1.82**	–	**n.d**
**95.**	**Caspase 13**	**C8854**	**1.82**	–	**1.75**
**96.**	**FXR2**	**F1554**	**1.82**	–	–
**97.**	**Caspase 10**	**C1229**	**1.81**	**1.75**	–
**98.**	**PKR**	**P0244**	–	–	**1.81**
**99.**	**hnRNP-C1/C2**	**R5028**	**1.80**	–	–
**100.**	**Importin α3**	**I9783**	**1.77**	**1.74**	–

**Table 2:** The proteins ranked 26–100 based on the detected fold change values after 3 h incubation of HUVEC with 10^−7^ M Ang-(1–7). The order of the numbers is oriented on the highest single value. Expression fold change lower than 1.5 is given in hyphen. Data that could not be detected is marked as n.d. Proteins marked in Italic show repeatedly identified differentially expressed proteins (RIDEPs). The mentioned dye indicates which dye the unstimulated sample was labeled with.

	**Protein**	**Antibody id**	**Cy3**	**Cy5**	**Cy5**
*26.*	*BID*	*B3183*	*2.85*	–	–
**27.**	**β-Tubulin**	**T5201**	**2.47**	**2.85**	**2.11**
*28.*	*Centrin*	*C7736*	*1.98*	–	*2.75*
**29.**	**p21**	**P1484**	**2.12**	–	**2.68**
**30.**	**FOXC2**	**F1054**	–	–	**2.67**
**31.**	**PIAS2**	**P9498**	–	**2.64**	**2.09**
**32.**	**Annexin VII**	**A4475**	–	**2.64**	**1.69**
**33.**	**Neurofibromin**	**N3662**	**1.59**	–	**2.64**
**34.**	**AOP1**	**A7674**	**n.d**	**2.64**	–
**35.**	**TRAIL**	**T9191**	–	**2.31**	**2.62**
**36.**	**Rab5**	**R7904**	**2.05**	–	**2.57**
**37.**	**GFI1**	**G6670**	**n.d**	**2.51**	**n.d**
**38.**	**DRAK1**	**D1314**	**2.48**	–	**1.76**
**39.**	**Cdk3**	**C9987**	**2.01**	**2.47**	**1.57**
**40.**	**ASPP2**	**A4480**	**1.77**	**2.44**	**n.d**
**41.**	**S100**	**S2532**	**2.40**	**1.68**	**2.03**
**42.**	**N-Cadherin**	**C2542**	**1.83**	–	**2.40**
**43.**	**Nitrotyrosin**	**N0409**	**1.74**	**1.5**	**2.39**
**44.**	**MADD**	**M5683**	**n.d**	**2.37**	**1.81**
**45.**	**hSNF5/INI1**	**H9912**	**1.56**	–	**2.33**
*46.*	*IKKα*	*I6139*	*1.52*	–	*2.33*
**47.**	**PRMT1**	**P6871**	**1.78**	–	**2.32**
**48.**	**DR3**	**D3563**	–	–	**2.32**
**49.**	**PP2A**	**P8109**	–	**2.31**	–
**50.**	**Connexin-32**	**C3470**	**1.73**	–	**2.30**
**51.**	**Tal**	**T1075**	**1.61**	–	**2.29**
**52.**	**BACE 1**	**B0806**	–	**2.29**	–
**53.**	**Sir2**	**S5313**	–	–	**2.27**
**54.**	**ARP3**	**A5979**	**1.74**	–	**2.27**
**55.**	**Striatin**	**S0696**	–	–	**2.26**
*56.*	*SMAD4*	*S3934*	*1.60*	–	*2.18*
**57.**	**Apaf1**	**A8469**	**2.18**	–	–
**58.**	**p57kip2**	**P2735**	**1.84**	**2.13**	**2.16**
**59.**	**Sirt1**	**S5196**	**1.82**	–	**2.16**
**60.**	**RICK**	**R9650**	**2.16**	**1.66**	–
**61.**	**FAK (pTyr577)**	**F8926**	**2.15**	–	**1.56**
**62.**	**FKHRL1**	**F2178**	–	**2.15**	–
**63.**	**Cyclin A**	**C4710**	**n.d**	**2.14**	**n.d**
**64.**	**BAP1**	**B9303**	–	**2.14**	–
**65.**	**MBD4**	**M9817**	–	–	**2.12**
**66.**	**MeCP2**	**M9317**	**1.66**	**1.63**	**2.10**
**67.**	**HDAC8**	**H6412**	**2.05**	–	**2.10**
**68.**	**MAPK14 (nonActivated)**	**M8432**	–	**2.09**	–
**69.**	**TOM22**	**T6319**	**1.54**	**1.66**	**2.08**
**70.**	**Annexin V**	**A8604**	**1.89**	–	**2.06**
**71.**	**c-Myc**	**M4439**	–	–	**2.06**
**72.**	**DEDAF**	**D3316**	**2.06**	–	–
**73.**	**eNOS**	**N9532**	**1.64**	–	**2.05**
**74.**	**RALAR**	**R8529**	–	**2.03**	**n.d**
**75.**	**H3 (Ac-Lys9, pSer10)**	**H0788**	–	**2.00**	**2.03**
**76.**	**TSG101**	**T5826**	**2.02**	**1.95**	–
**77.**	**Dystrophin**	**D8168**	**1.66**	–	**2.02**
**78.**	**Connexin 43**	**C8093**	–	–	**2.01**
**79.**	**p63**	**P3362**	**1.89**	–	**1.96**
**80.**	**Protein Kinase Bα**	**P2482**	–	–	**1.96**
**81.**	**Calretinin**	**C7479**	–	**1.96**	–
**82.**	**Coilin**	**C1862**	–	**1.94**	–
*83.*	*MyD88*	*M9934*	–	–	*1.91*
**84.**	**ROCK 2**	**R8653**	**1.77**	–	**1.90**
**85.**	**I-Afadin**	**A0349**	**1.52**	–	**1.90**
**86.**	**Connexin 43**	**C6219**	**1.56**	–	**1.89**
**87.**	**α-Actinin**	**A5044**	**1.58**	–	**1.88**
**88.**	**E2F1**	**E9026**	**1.88**	**1.85**	–
**89.**	**Chk2**	**C9233**	**1.88**	–	**1.84**
**90.**	**Importin α1**	**I9658**	**1,87**	–	–
**91.**	**F1Aα**	**F3428**	–	**1.86**	–
**92.**	**SUMO1**	**S5446**	–	**1.85**	–
**93.**	**ASPP1**	**A4355**	**1.84**	–	–
**94.**	**SynCAM**	**S4945**	–	**1.84**	–
**95.**	**Chk1**	**C9358**	**1.80**	**1.51**	**1.70**
**96.**	**Sp1**	**S9809**	**1.80**	–	**1.60**
**97.**	**Pyk2 (pTyr579)**	**P7114**	**n.d**	**1.80**	**n.d**
**98.**	**RIP**	**R8274**	**1.63**	–	**1.79**
**99.**	**Transportin 1**	**T0825**	**1.54**	–	**1.77**
**100.**	**GADD153**	**G6916**	**1.56**	–	**1.76**

**Table 3:** The proteins ranked 26–31 based on the detected fold change values after 6 h incubation of HUVEC with 10^−7^ M Ang-(1–7). The order of the numbers is oriented on the highest single value. Expression fold change lower than 1.5 is given in hyphen. Data that could not be detected is marked as n.d. Proteins marked in Italic show repeatedly identified differentially expressed proteins (RIDEPs). The mentioned dye indicates which dye the unstimulated sample was labeled with.

	**Protein**	**Antibody id**	**Cy3**	**Cy5**	**Cy5**
**26.**	**E2F1**	**E9026**	**1.89**	–	–
**27.**	**Zip Kinase**	**Z0134**	–	**1.87**	–
*28.*	*BID*	*B3183*	–	–	*1.85*
**29.**	**Cyclin D1**	**C7464**	–	**1.85**	–
**30.**	**Nerve Growth Factor β**	**N3279**	**1.81**	**n.d**	–
**31.**	**HDAC7**	**H2537**	**1.78**	**1.57**	–

**Table 4:** The proteins ranked 26–86 based on the detected fold change values after 9 h incubation of HUVEC with 10^−7^ M Ang-(1–7). The order of the numbers is oriented on the highest single value. Expression fold change lower than 1.5 is given in hyphen. Data that could not be detected is marked as n.d. Proteins marked in Italic show repeatedly identified differentially expressed proteins (RIDEPs). The mentioned dye indicates which dye the unstimulated sample was labeled with.

	**Protein**	**Antibody id**	**Cy3**	**Cy5**	**Cy5**
**26.**	**DR3**	**D3563**	**2.67**	–	–
**27.**	**DNase I**	**D0188**	**2.65**	–	–
**28.**	**Nitrotyrosin**	**N0409**	**2.61**	**2.27**	–
**29.**	**NGFR**	**N3908**	**n.d**	**2.57**	–
**30.**	**MDC1**	**M2444**	**2.55**	–	–
**31.**	**p120**	**P1870**	**2.54**	–	–
**32.**	**mTor**	**T2949**	–	**2.48**	–
*33.*	*BID*	*B3183*	*2.43*	*1.71*	*2.14*
**34.**	**MDM2**	**M4308**	**2.42**	–	–
**35.**	**WAVE**	**W0392**	**2.38**	–	–
**36.**	**MAP1**	**M6783**	**n.d**	**2.38**	–
**37.**	**c-Raf (pSer621)**	**R1151**	**n.d**	**2.37**	**n.d**
**38.**	**TGF β**	**T9429**	**n.d**	**1.41**	**2.31**
**39.**	**DR4**	**D3813**	**2.28**	–	–
**40.**	**HDAC6**	**H2287**	**2.26**	–	–
**41.**	**Desmosomal Protein**	**D1286**	**2.24**	–	–
**42.**	**Calnexin**	**C4731**	**2.21**	–	–
**43.**	**FGF9**	**F1672**	**2.19**	–	–
**44.**	**Coilin**	**C1862**	**2.18**	**1.61**	–
**45.**	**Phospholipase Cγ1**	**P8104**	–	**2.17**	–
**46.**	**APRIL**	**A1851**	**2.17**	**n.d**	**n.d**
**47.**	**TBP**	**T1827**	–	**2.08**	**2.16**
**48.**	**DRAK1**	**D1314**	**n.d**	**1.81**	**2.13**
**49.**	**UCHL1**	**U5258**	**2.12**	–	–
**50.**	**Parkin**	**P6248**	**2.09**	–	–
**51.**	**PIASγ**	**P0104**	**2.09**	–	–
**52.**	**GFI1**	**G6670**	**2.07**	–	–
**53.**	**HDAC5**	**H4538**	**2.07**	–	–
**54.**	**Protein Kinase Cβ2**	**P3203**	**1.89**	**2.05**	–
**55.**	**Apaf1**	**A8469**	**2.03**	–	–
**56.**	**MRP2**	**M3692**	**n.d**	**2.01**	–
**57.**	**Neurabin II**	**N5037**	**1.99**	–	–
**58.**	**AP1**	**A5968**	**1.98**	–	–
**59.**	**p19**	**P4354**	**1.97**	–	–
**60.**	**hABH2**	**A8228**	**1.96**	–	–
**61.**	**E2F1**	**E9026**	**1.95**	**1.87**	**1.63**
**62.**	**E2F6**	**E1532**	**n.d**	**n.d**	**1.95**
**63.**	**PRMT2**	**P0748**	–	–	**1.95**
**64.**	**TAP**	**T1076**	**1.94**	–	–
**65.**	**PRMT1**	**P6871**	**n.d**	–	**1.89**
**66.**	**ε-Tubulin**	**T1323**	**1.89**	–	–
**67.**	**MDMX**	**M0445**	**1.88**	**1.52**	–
**68.**	**Paxillin**	**P1093**	**1.88**	–	–
**69.**	**Filamin**	**F1888**	**1.88**	**1.69**	–
**70.**	**Caldesmon**	**C6542**	**1.88**	**1.85**	–
**71.**	**p34**	**C3085**	**1.87**	–	–
**72.**	**JNK**	**J4500**	**1.86**	**1.6**	–
**73.**	**Survivin**	**S8191**	**1.86**	–	–
**74.**	**Melanocortin 3**	**M4937**	**1.86**	–	–
**75.**	**H3 (pSer10)**	**H6409**	**1.85**	–	**1.86**
**76.**	**Sir2**	**S5313**	**1.50**	**1.84**	–
**77.**	**Ciliated Cell Marker**	**C5867**	**1.84**	–	–
**78.**	**Collagen Type IV**	**C1926**	**1.82**	–	–
**79.**	**MAPK14**	**M8432**	**1.81**	–	–
**80.**	**FANCD2**	**F0305**	**1.81**	–	–
**81.**	**Syntaxin 8**	**S8945**	**1.81**	–	–
**82.**	**S6 Kinase**	**S4047**	–	–	**1.80**
**83.**	**HDAC10**	**H3413**	**1.64**	**1.76**	–
**84.**	**Chk1**	**C9358**	**1.52**	–	**1.60**
**85.**	**Bcl-x**	**B9304**	**1.59**	**1.53**	–
**86.**	**Aly**	**A9979**	**1.59**	**1.57**	–

**Table 5:** Metabolic pathways ranked position 11–25 (bold) using the *p*-values associated by the IPA software to each of the different antibody microarray sets (A: 1 h; B: 3 h; C: 6 h; D: 9 h). For completion, the ranking of the first ten pathways are also listed (in Italic). The ratio states the number of proteins detected in the microarray versus the total number of proteins being part of the particular pathway.

**A)**			
	**Pathway**	**1 h**	**Ratio**
		***p*-Value**	
*1.*	*Molecular Mechanisms of Cancer*	*3.67E–11*	*17/379 (4.5%)*
*2.*	*p53 Signaling*	*6.57E–09*	*9/96 (9.4%)*
*3.*	*Glucocorticoid Receptor Signaling*	*9.70E–09*	*13/295 (4.4%)*
*4.*	*Chemokine Signaling*	*2.44E–07*	*7/73 (9.6%)*
*5.*	*Cyclins and Cell Cycle regulation*	*6.24E–07*	*7/89 (7.9%)*
*6.*	*PI3K/AKT Signaling*	*7.24E–07*	*8/140 (5.7%)*
*7.*	*ATM Signaling*	*7.59E–07*	*6/54 (11.1%)*
*8.*	*VEGF Signaling*	*8.91E–07*	*7/99 (7.1%)*
*9.*	*Apoptosis Signaling*	*1.26E–06*	*7/96 (7.3%)*
*10.*	*Death Receptor Signaling*	*1.58E–06*	*6/95 (9.9%)*
**11.**	**Chronic Myeloid Leukemia Signaling**	**2.00E–06**	**7/105 (6.7%)**
**12.**	**ERK/MAPK Signaling**	**2.94E–06**	**9/204 (4.4%)**
**13.**	**Cholecystokinin/Gastrin-mediated Signaling**	**3.62E–06**	**7/106 (6.6%)**
**14.**	**Parkinson׳s Signaling**	**4.80E–06**	**4/18 (22.2%)**
**15.**	**Pancreatic Adenocarcinoma Signaling**	**5.01E–06**	**7/119 (5.9%)**
**16.**	**CCR5 Signaling in Macrophages**	**5.92E–06**	**6/94 (6.4%)**
**17.**	**PTEN Signaling**	**6.31E–06**	**7/124 (5.6%)**
**18.**	**PDGF Signaling**	**6.80E–06**	**6/79 (7.6%)**
**19.**	**Renin-Angiotensin Signaling**	**7.30E–06**	**7/126 (5.6%)**
**20.**	**PI3K Signaling in B Lymphocytes**	**2.40E–05**	**7/143 (4.9%)**
**21.**	**Protein Kinase A Signaling**	**2.76E–05**	**10/328 (3.0%)**
**22.**	**Breast Cancer Regulation by Stathmin1**	**3.17E–05**	**8/210 (3.8%)**
**23.**	**B Cell Receptor Signaling**	**3.40E–05**	**7/156 (4.5%)**
**24.**	**IGF-1 Signaling**	**4.50E–05**	**6/107 (5.6%)**
**25.**	**IL-15 Signaling**	**5.18E–05**	**5/67 (7.5%)**

**B)**			
	**Pathway**	**3 h**	**Ratio**
		***p*-Value**	
*1.*	*Molecular Mechanisms of Cancer*	*1.20E−22*	*32/379 (8.4%)*
*2.*	*p53 Signaling*	*3.00E−20*	*19/96 (19.7%)*
*3.*	*Chronic Myeloid Leukemia Signaling*	*1.59E−14*	*15/105 (14.3%)*
*4.*	*Cyclins and Cell Cycle regulation*	*2.70E−14*	*14/89 (15.7%)*
*5.*	*Death Receptor Signaling*	*3.51E−13*	*12/65 (18.5%)*
*6.*	*Cell Cycle: G1/S Checkpoint regulation*	*3.98E−12*	*11/61 (18.0%)*
*7.*	*PTEN Signaling*	*3.16E−11*	*13/124 (10.5%)*
*8.*	*VEGF Signaling*	*5.01E−11*	*12/99 (12.1%)*
*9.*	*PI3K/AKT Signaling*	*1.58E−10*	*13/140 (9.3%)*
*10.*	*Huntington׳s Disease Signaling*	*7.94E−10*	*16/238 (6.7%)*
**11.**	**TNFR1 Signaling**	**1.94E−09**	**9/53 (17.0%)**
**12.**	**Aryl Hydrocarbon Receptor Signaling**	**2.24E−09**	**13/159 (8.2%)**
**13.**	**Apoptosis Signaling**	**2.51E−09**	**11/96 (11.5%)**
**14.**	**IL-8 Signaling**	**3.80E−09**	**14/193 (7.3%)**
**15.**	**Small Cell Lung Cancer Signaling**	**3.98E−09**	**18/89 (11.2%)**
**16.**	**ATM Signaling**	**5.01E−09**	**9/54 (16.7%)**
**17.**	**Glioma Signaling**	**6.31E−09**	**11/112 (9.8%)**
**18.**	**Role of PKR in Interferon Induction and Antiviral Response**	**1.22E−08**	**8/46 (17.4%)**
**19.**	**Pancreatic Adenocarcinoma Signaling**	**2.00E−08**	**11/119 (9.2%)**
**20.**	**TWEAK Signaling**	**4.66E−08**	**7/39 (17.9%)**
**21.**	**Glucocorticoid Receptor Signaling**	**5.01E−08**	**15/295 (5.1%)**
**22.**	**Myc Mediated Apoptosis Signaling**	**5.08E−08**	**8/61 (13.1%)**
**23.**	**Type I Diabetes Mellitus Signaling**	**5.15E−08**	**10/121 (8.3%)**
**24.**	**Induction of Apoptosis by HIV1**	**5.22E−08**	**8/66 (12.1%)**
**25.**	**Hereditary Breast Cancer Signaling**	**5.25E−08**	**10/129 (7.8%)**

**C)**			
	**Pathway**	**6 h**	**Ratio**
		***p*-Value**	
*1.*	*Molecular Mechanisms of Cancer*	*1.33E−09*	*3/379 (2.4%)*
*2.*	*Small Cell Lung Cancer Signaling*	*7.65E−08*	*5/89 (5.6%)*
*3.*	*Cyclins and Cell Cycle regulation*	*1.31E−07*	*5/89 (5.6%)*
*4.*	*VEGF Signaling*	*2.14E−07*	*5/99 (5.1%)*
*5.*	*p53 Signaling*	*2.99E−07*	*5/96 (5.2%)*
*6.*	*Chronic Myeloid Leukemia Signaling*	*3.16E−07*	*5/105 (4.8%)*
*7.*	*Glioma Signaling*	*3.98E−07*	*5/112 (4.5%)*
*8.*	*GM-CSF Signaling*	*3.16E−06*	*4/67 (6.0%)*
*9.*	*IL-8 Signaling*	*6.31E−06*	*5/193 (2.6%)*
*10.*	*HGF Signaling*	*2.00E−05*	*4/105 (3.8%)*
**11.**	**Huntington׳s Disease Signaling**	**2.19E−05**	**5/238 (2.1%)**
**12.**	**Pancreatic Adenocarcinoma Signaling**	**2.29E−05**	**4/119 (3.4%)**
**13.**	**PTEN Signaling**	**2.69E−05**	**4/124 (3.2%)**
**14.**	**14-3-3-mediated Signaling**	**3.28E−05**	**4/120 (3.3%)**
**15.**	**PI3K/AKT Signaling**	**3.31E−05**	**4/140 (2.9%)**
**16.**	**p70S6K Signaling**	**4.15E−05**	**4/130 (3.1%)**
**17.**	**Melanoma Signaling**	**4.41E−05**	**3/46 (6.5%)**
**18.**	**Cell Cycle: G1/S Checkpoint regulation**	**8.91E−05**	**3/61 (4.9%)**
**19.**	**Induction of Apoptosis by HIV1**	**1.28E−04**	**3/66 (4.5%)**
**20.**	**IL-15 Signaling**	**1.36E−04**	**3/67 (4.5%)**
**21.**	**Retinoic acid Mediated Apoptosis Signaling**	**1.44E−04**	**3/68 (4.4%)**
**22.**	**Non-Small Cell Lung Cancer Signaling**	**1.83E−04**	**3/79 (3.8%)**
**23.**	**Chemokine Signaling**	**6.31E−04**	**3/73 (4.1%)**
**24.**	**ERK/MAPK Signaling**	**6.37E−04**	**4/204 (2.0%)**
**25.**	**Integrin Signaling**	**6.41E−04**	**4/210 (1.9%)**

**D)**			
	**Pathway**	**9 h**	**Ratio**
		***p*-Value**	
*1.*	*p53 Signaling*	*1.73E−19*	*15/96 (15.6%)*
*2.*	*Molecular Mechanisms of Cancer*	*1.14E−15*	*19/379 (5.0%)*
*3.*	*Chronic Myeloid Leukemia Signaling*	*1.71E−14*	*12/105 (11.4%)*
*4.*	*ATM Signaling*	*3.94E−14*	*10/54 (18.5%)*
*5.*	*Huntington׳s Disease Signaling*	*1.48E−12*	*14/238 (5.9%)*
*6.*	*Cyclins and Cell Cycle regulation*	*2.51E−12*	*10/89 (11.2%)*
*7.*	*Glioma Signaling*	*3.16E−11*	*10/112 (8.9%)*
*8.*	*Role of NFAT in Cardiac Hypertrophy*	*6.31E−11*	*12/208 (5.8%)*
*9.*	*Pancreatic Adenocarcinoma Signaling*	*7.94E−11*	*10/119 (8.4%)*
*10.*	*Hereditary Breast Cancer Signaling*	*2.00E−10*	*10/129 (7.8%)*
**11.**	**B Cell Receptor Signaling**	**2.02E−09**	**10/156 (6.4%)**
**12.**	**Glucocorticoid Receptor Signaling**	**1.26E−08**	**12/295 (4.1%)**
**13.**	**PI3K/AKT Signaling**	**1.58E−08**	**9/140 (6.4%)**
**14.**	**FGF Signaling**	**1.69E−08**	**8/90 (8.9%)**
**15.**	**Cell Cycle: G1/S Checkpoint regulation**	**1.91E−08**	**7/61 (11.5%)**
**16.**	**Neuregulin Signaling**	**1.92E−08**	**8/102 (7.8%)**
**17.**	**HGF Signaling**	**2.75E−08**	**8/105 (7.6%)**
**18.**	**IGF-1 Signaling**	**3.16E−08**	**8/107 (7.5%)**
**19.**	**Erythropoietin Signaling**	**3.58E−08**	**7/78 (9.0%)**
**20.**	**Glioblastoma Multiforme Signaling**	**4.60E−08**	**9/164 (5.5%)**
**21.**	**Neurotrophin/TRK Signaling**	**6.70E−08**	**7/77 (9.1%)**
**22.**	**FLT3 Signaling in Hematopoietic Progenitor Cells**	**8.61E−08**	**7/74 (9.5%)**
**23.**	**14-3-3-mediated Signaling**	**9.75E−08**	**8/120 (6.7%)**
**24.**	**Acute Phase Response Signaling**	**1.11E−07**	**9/178 (5.1%)**
**25.**	**Cardiac Hypertrophy Signaling**	**1.61E−07**	**10/245 (4.1%)**
